# Resistance exercise acutely elevates dynamic cerebral autoregulation gain

**DOI:** 10.14814/phy2.15676

**Published:** 2023-04-26

**Authors:** Oliver J. Smail, Daniel J. Clarke, Qais Al‐Alem, William Wallis, Alan R. Barker, Jonathan D. Smirl, Bert Bond

**Affiliations:** ^1^ Exeter Head Impacts, Brain Injury and Trauma (ExHIBIT), Public Health and Sport Sciences University of Exeter Exeter UK; ^2^ Children's Health and Exercise Research Centre University of Exeter Exeter UK; ^3^ Faculty of Kinesiology University of Calgary Calgary Alberta Canada; ^4^ Alberta Children's Hospital Reach Institute University of Calgary Calgary Alberta Canada; ^5^ Hotchkiss Brain Institute University of Calgary Calgary Alberta Canada; ^6^ Libin Cardiovascular Institute of Alberta University of Calgary Alberta Canada; ^7^ Cerebrovascular Concussion Lab University of Calgary Calgary Alberta Canada

**Keywords:** blood pressure, cerebral blood flow, cerebrovascular, pressure‐flow relationship, squats

## Abstract

Dynamic cerebral autoregulation (dCA) describes the regulation of cerebral blood flow (CBF) in response to fluctuations in systemic blood pressure (BP). Heavy resistance exercise is known to induce large transient elevations in BP, which are translated into perturbations of CBF, and may alter dCA in the immediate aftermath. This study aimed to better quantify the time course of any acute alterations in dCA after resistance exercise. Following familiarisation to all procedures, 22 (14 male) healthy young adults (22 ± 2 years) completed an experimental trial and resting control trial, in a counterbalanced order. Repeated squat‐stand manoeuvres (SSM) at 0.05 and 0.10 Hz were used to quantify dCA before, and 10 and 45 min after four sets of ten repetition back squats at 70% of one repetition maximum, or time matched seated rest (control). Diastolic, mean and systolic dCA were quantified by transfer function analysis of BP (finger plethysmography) and middle cerebral artery blood velocity (transcranial Doppler ultrasound). Mean gain (*p* = 0.02; *d* = 0.36) systolic gain (*p* = 0.01; *d* = 0.55), mean normalised gain (*p* = 0.02; *d* = 0.28) and systolic normalised gain (*p* = 0.01; *d* = 0.67) were significantly elevated above baseline during 0.10 Hz SSM 10‐min post resistance exercise. This alteration was not present 45 min post‐exercise, and dCA indices were never altered during SSM at 0.05 Hz. dCA metrics were acutely altered 10 min post resistance exercise at the 0.10 Hz frequency only, which indicate changes in the sympathetic regulation of CBF. These alterations recovered 45 min post‐exercise.

## Graphical abstract

Resistance exercise can provide a substantial increase in blood pressure, and thus a significant autoregulatory challenge. Our understanding of how resistance exercise acutely influences the regulation of brain blood flow to changes in blood pressure is poor. Our paper demonstrate that this relationship is disturbed 10, but not 45, minutes after 4 sets of weighted squats in healthy young adults.

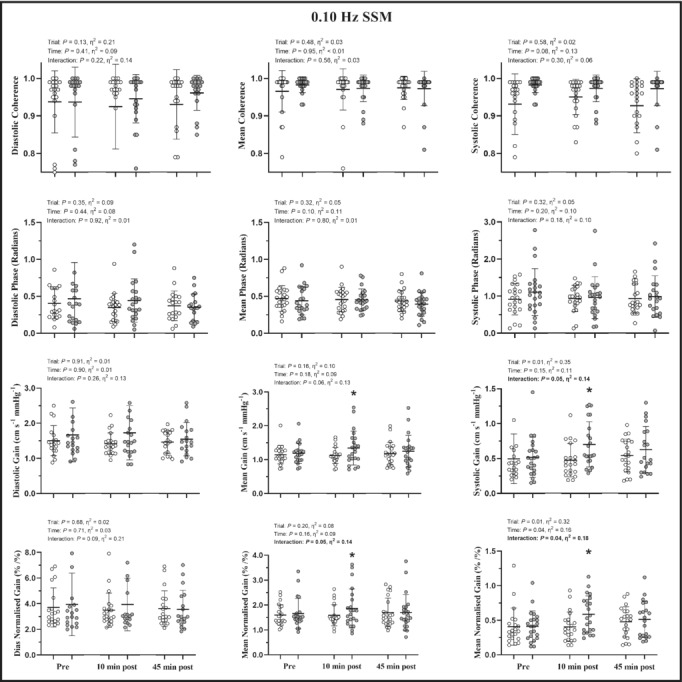

## 1 INTRODUCTION

Dynamic cerebral autoregulation (dCA) describes the physiological process of defending cerebral blood flow (CBF) despite rapid fluctuations in systemic blood pressure (BP) (Aaslid et al., 1989; Brassard et al., 2021). Effective dCA is thought to be a component of good cerebrovascular health, with evidence of altered dCA in various neuropathological states including Alzheimer's disease (Claassen & Zhang, 2011), stroke (Castro et al., 2018) and following traumatic brain injury (Sviri et al., 2009). Effective dCA is also associated with better neurological outcomes following stroke and has been advocated for use in monitoring stroke recovery (Castro et al., 2017).

Resistance exercise is a popular leisure‐time activity, which is recommended by national and international public health guidelines (Piercy et al., 2018) and is a common training component in competitive sport. BP can be acutely elevated to very high levels during resistance exercise; BP values of 480/350 mmHg have been reported during bilateral leg press exercise (MacDougall et al., 1985). Dampening such large and rapid fluctuations in BP, in order to protect the cerebrovasculature from a potential overperfusion injury, present a substantial autoregulatory challenge (Perry et al., 2020). The extent of such BP elevations is dependent upon the intensity and type of movement involved, and not all resistance exercise is performed under maximal exertion. However, it has recently been demonstrated that dCA is altered in young healthy females, but not males, even during light resistance exercise whereby mean arterial pressure (MAP) is raised by ~15 mmHg (Newel et al., 2022).

Presently, dCA has been shown to be acutely altered in the *immediate* aftermath (<90 s) of resistance exercise (Koch et al., 2005; Perry et al., 2014). However, whether dCA remains altered beyond these initial seconds postexercise remains unexplored, and little insight is available regarding whether such changes reflect alterations in sympathetic or myogenic contributions to dCA. Given that resistance exercise is advocated for improved long‐term health outcomes (McLeod et al., 2019; Piercy et al., 2018), the purpose of this study was to better identify the acute time course of any changes in dCA following a single resistance exercise session using contemporary methodological guidelines (Smirl et al., 2015).

## 2 METHODS

Ethics approval was obtained from the University of Exeter Sport and Health Sciences Ethics Committee (2019/M/10), and participants provided written informed consent (which included the use of data in future publications) prior to any data collection. The study was conducted in accordance with 1964 Declaration of Helsinki. Twenty‐two young, healthy participants (22 ± 2y; 14 male) with a wide range of resistance training experience (from none to 6 years of training) were recruited from the University of Exeter (Table 1). Exclusion criteria included injury likely to impair the ability to safely perform a loaded barbell squat, known conditions likely to increase the risk of syncope induced by fluctuating blood pressure, a history or neurological, cerebrovascular, cardiorespiratory or musculoskeletal complications and any history of performance‐enhancing drug use, specifically anabolic agents.

**TABLE 1:** Participant characteristics.

	Mean ± SD	Range
N	22	‐
Male:Female	14:8	‐
Age (years)	22 ± 2	19–25
Mass (kg)	77.5 ± 16.6	54.0–109.0
Stature (m)	1.74 ± 0.09	1.54–1.91
BMI (kg.m^−1^)	25.2 ± 3.5	19.8–31.8
1RM (kg)	117 ± 37	66–211
70% 1RM (kg)	82 ± 26	46–148
Training history (months)	33 ± 29	0–96

Abbreviations: 1RM, one repetition maximum; BMI, body mass index; N, number of participants; SD, standard deviation.

Participants completed three total visits to the laboratory, each separated by at least 2 days, with most visits being separated by an average of 1 week and none more than 2 weeks. Participants were instructed to avoid food or caffeine for at least 2 hours prior to arrival, and not to complete strenuous exercise in the daytime preceding their visit to control for potential confounding effects on BP or dCA (Burma, Copeland, Macaulay, Khatra, & Smirl, 2020a).

On the first visit, stature (Seca stadiometer SEC‐225, Seca, Hamburg, Germany) and body mass (Hampel XWM‐150 K, Hampel Electronics Co., Taiwan) were recorded to the nearest 0.1 cm and 0.1 kg, respectively. Participants were asked for their resistance training history in months. All participants were then familiarized to the techniques involved in dCA data acquisition and the back squat movement, prior to the assessment of their one repetition maximum (1RM). All squats were completed using a safety squat bar to simplify the movement for inexperienced participants. During the loaded back squats, participants were instructed to squat to their personal maximum depth (typically thighs parallel to the ground) before standing back up under supervision of experienced spotters.

**FIGURE 1:**
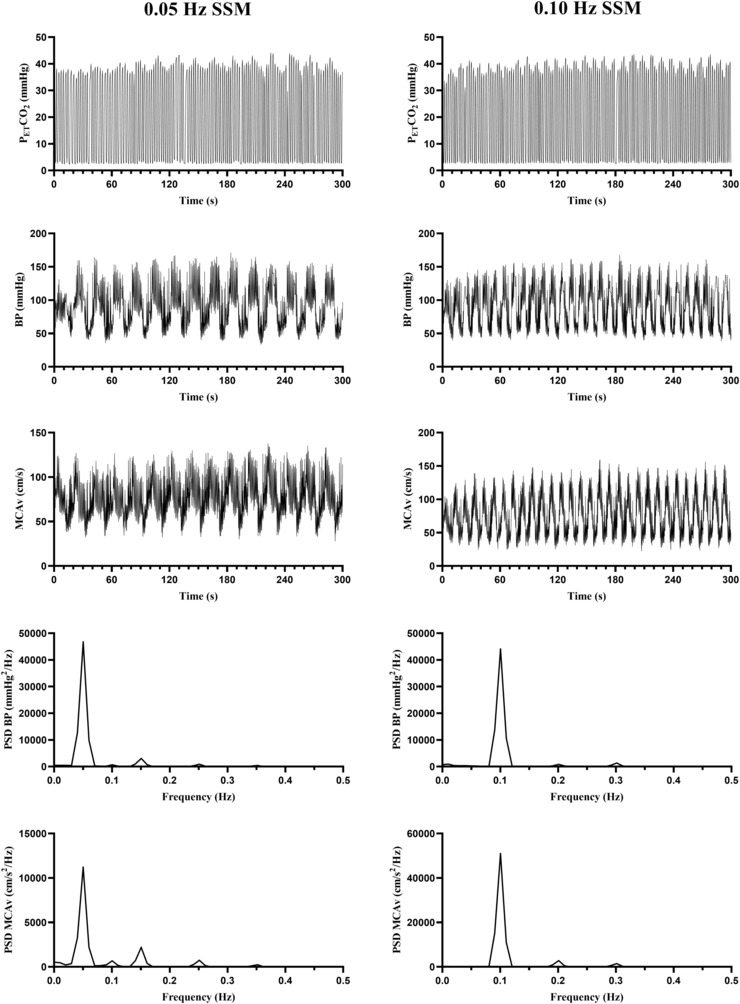
Representative data from a single participant during the squat‐stand maneuvers (SSM) at both 0.05 Hz (20 s squat, 20 s stand) and 0.10 Hz (10 s squat, 10 s stand) frequencies. P_ET_CO_2_, partial pressure of end‐tidal carbon dioxide; BP, blood pressure; MCAv, blood velocity in the middle cerebral artery; PSD, power spectrum density.

Visits two and three consisted of the intervention and control trials and completed in a counterbalanced order between participants. dCA was assessed using repeated squat‐stand maneuvers (SSM), in line with current recommendations (Panerai et al., 2023; Smirl et al., 2015). Participants underwent two separate 5‐min SSM at 0.05 Hz (squat for 20 s and stand for 20 s) and 0.10 Hz (squat for 10 s and stand for 10 s), achieving a ~ 90° knee‐bend with each squat. Representative data from one participant performing the two SSM are provided in Figure 1. The frequency of the two SSM was counterbalanced between participants. Participants were given 2‐min seated recovery between tests and were instructed to stand still for 1 min prior to initiation of the remaining SSM to allow recovery from initial orthostatic hypotension (Narayanan et al., 2001). Resting mean arterial pressure (MAP) of the brachial artery was determined using an automated cuff device (Carescape V100, Dinamap, GE Healthcare, UK) prior to performing the SSM. Resting values for the partial pressure of end‐tidal CO_2_ and middle cerebral artery blood flow velocity (MCA_V_) were calculated by averaging the final 30 s of standing data prior to the SSM. Next, participants either completed four sets of 10 repetition back squats at 70% 1RM over a 15‐min duration (intervention trial), which may reflect typical training practices, or sat for the same duration (15 min; control trial). Participants were given 3 min of seated recovery between back squat sets. All outcomes were subsequently remeasured 10‐ and 45‐min postexercise (intervention) or sitting (control), as acute hemodynamic and vascular alterations postexercise may be transient in nature (DeVan et al., 2005; Lefferts et al., 2014), and because these time points likely reflect a period where individuals would be performing further resistance exercise as part of a single training session.

CBF_V_ was determined by the use of transcranial Doppler ultrasonography. The right middle cerebral artery (MCA) was insonated using a 2‐MHz probe (Doppler‐BoxX, DWL, Germany) and subsequently locked into place using an adjustable headset (DWL, Germany) to determine MCA_V_. MCA insonation was confirmed by the identification of the anterior cerebral artery bifurcation or momentary unilateral carotid occlusion (Willie et al., 2011). Beat‐to‐beat BP was measured via finger plethysmography, calibrated to participant characteristics (age, stature, body mass and sex), and height offset was employed to correct for pressure changes between the finger location and the blood pressure at heart level (Portapres, Finapres Medical Systems, Netherlands). A 3‐lead electrocardiogram was used for R‐R interval calibration. The partial pressure of end‐tidal CO_2_ (P_ET_CO_2_) was measured breath‐by‐breath using an online gas analyzer (ML206, ADInstruments) calibrated to known gas concentrations prior to each assessment. All data were sampled at a frequency of 1000 Hz (PowerLab 8/30 ML880, ADInstruments) during dCA acquisition and stored for subsequent offline analysis with commercially available software (LabChart version 7.1, ADInstruments).

Diastolic, mean and systolic dCA was determined for each SSM and quantified using transfer function analysis of BP (input) and MCA_V_ (output), in accordance with contemporary guidelines (Panerai et al., 2023), using dedicated software (Elucimed, Ensemble‐R, New Zealand), which incorporated R‐R interval calibration. Diastolic and systolic components were analyzed separately from the mean due to evidence that dCA may be differentially regulated across the cardiac cycle (Newel et al., 2022; Ogoh et al., 2005; Smirl et al., 2018). Output summaries of coherence, phase, gain and normalized gain (%.mmHg^−1^) for 0.05 Hz and 0.10 Hz SSM were collated for later statistical analysis, in order to provide insight regarding myogenic and sympathetic mechanisms of regulation, respectively (Hamner et al., 2010). These outcomes were calculated at the point estimates of the SSM frequency, verified by visual inspection of BP and MCA_V_ power spectrum densities (Smirl et al., 2015), as demonstrated in Figure 1. There was no evidence of phase wrap‐around at any point estimate (Claassen et al., 2016).

As the recruited participants were heterogenous for resistance exercise experience, a preliminary ANOVA model was used to determine whether stratifying based on training status and 1RM performance had an effect on the study outcomes. However, no significant interactions were identified. Additionally, no main or interaction effect was observed for sex, in line with previous work (Burma, Copeland, Macaulay, Khatra, & Smirl, 2020a). Therefore, participants were subsequently pooled into a single group for postexercise time‐by‐trial interaction analyses. Fisher's least significant difference post hoc analysis was applied to identify any significant differences, and statistical significance was accepted when *p* < 0.05. ANOVA main and interaction effects are presented as p values and partial eta squared (η_p_^2^), interpreted as small (<0.06), moderate (0.06 < η_p_^2^ < 0.14) and large (>0.14) effects. The magnitude of post hoc pairwise comparisons was explored using standardized effect sizes (Cohen's *d*) and interpreted as small (<0.20), moderate (0.20–0.50) and large (>0.50) (Cohen, 1988). All statistical analyses were completed using SPSS (IBM Corp, USA, v26.0).

## 3 RESULTS

Each participant successfully completed all testing sessions. However, due to a technical issue, P_ET_CO_2_ data are only available in a subsample of 10 participants.

**FIGURE 2:**
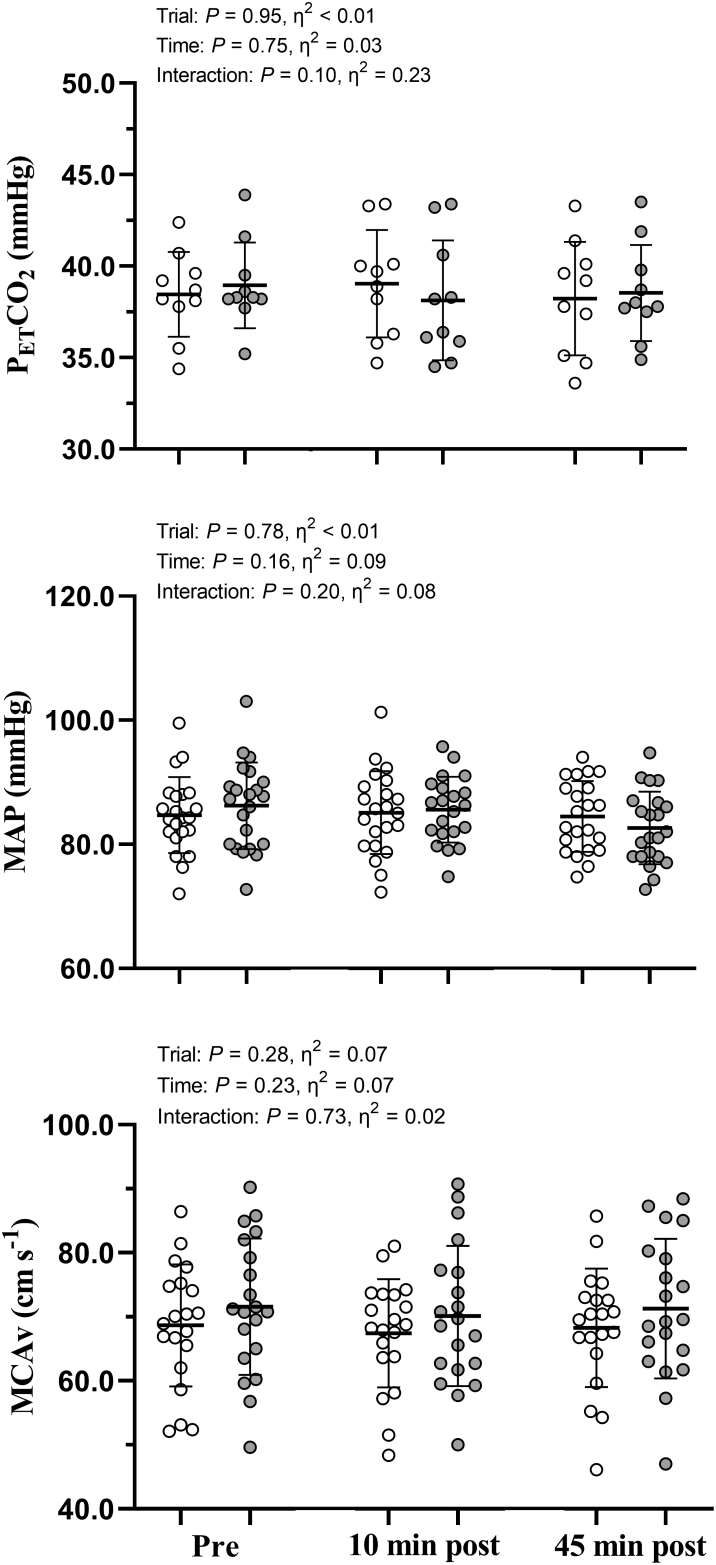
Resting end‐tidal carbon dioxide concentrations (P_ET_CO_2_), mean arterial blood pressure (MAP), and blood velocity in the middle cerebral artery (MCAv) during the 1 min of standing immediately preceding the squat‐stand maneuvers. Data are presented as individual values, the mean (horizontal line) and standard deviation (error bars). The control trial (time‐matched seated rest) is presented in white. The gray circles denote the resistance exercise intervention trial (4 sets of 10 repetition back squats at 70% one repetition maximum). Note that *n* = 10 for P_ET_CO_2_ due to equipment failure. Repeated measures ANOVA failed to reveal any significant time‐by‐trial interactions *p* > 0.10; η_p_^2^ < 0.23.

No significant time‐by‐trial interactions were identified for resting measures of P_ET_CO_2_, MAP or MCA_V_ (*p* > 0.10; η_p_^2^ < 0.23 for all, Figure 2). Mean coherence was >0.90 across all phases of the cardiac cycle during 0.05 and 0.10 Hz SSM. P_ET_CO_2_ was never altered during SSM at 0.05 (*p* = 0.61; η_p_^2^ = 0.07) and 0.10 Hz (*p* = 0.84; η_p_^2^ = 0.02) and always remained within a range of ±7.5 mmHg throughout each test.

**FIGURE 3:**
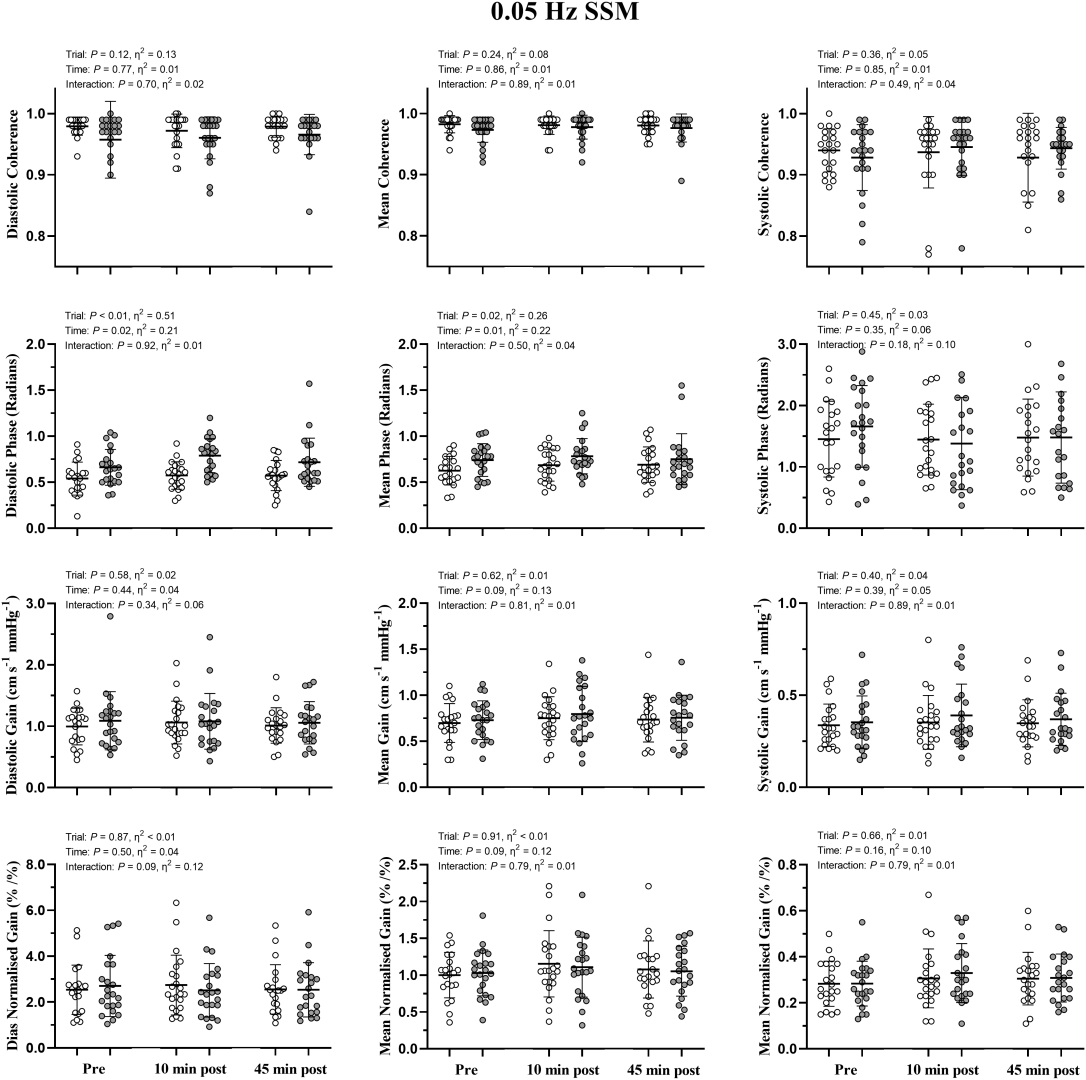
dCA metrics during squat‐stand maneuvers (SSM) at 0.05 Hz pre‐intervention, 10‐ and 45‐minute postintervention. Data are presented as individual values, the mean (horizontal line) and standard deviation (error bars). The control trial (time‐matched seated rest) is presented in white. The gray circles denote the resistance exercise intervention trial (4 sets of 10 repetition back squats at 70% one repetition maximum). Please note the differences in y axes within an outcome, but across the cardiac cycle. Repeated measures ANOVA failed to reveal a significant time‐by‐trial interaction for any dCA metric *p* > 0.09; η_p_^2^ < 0.12.

**FIGURE 4:**
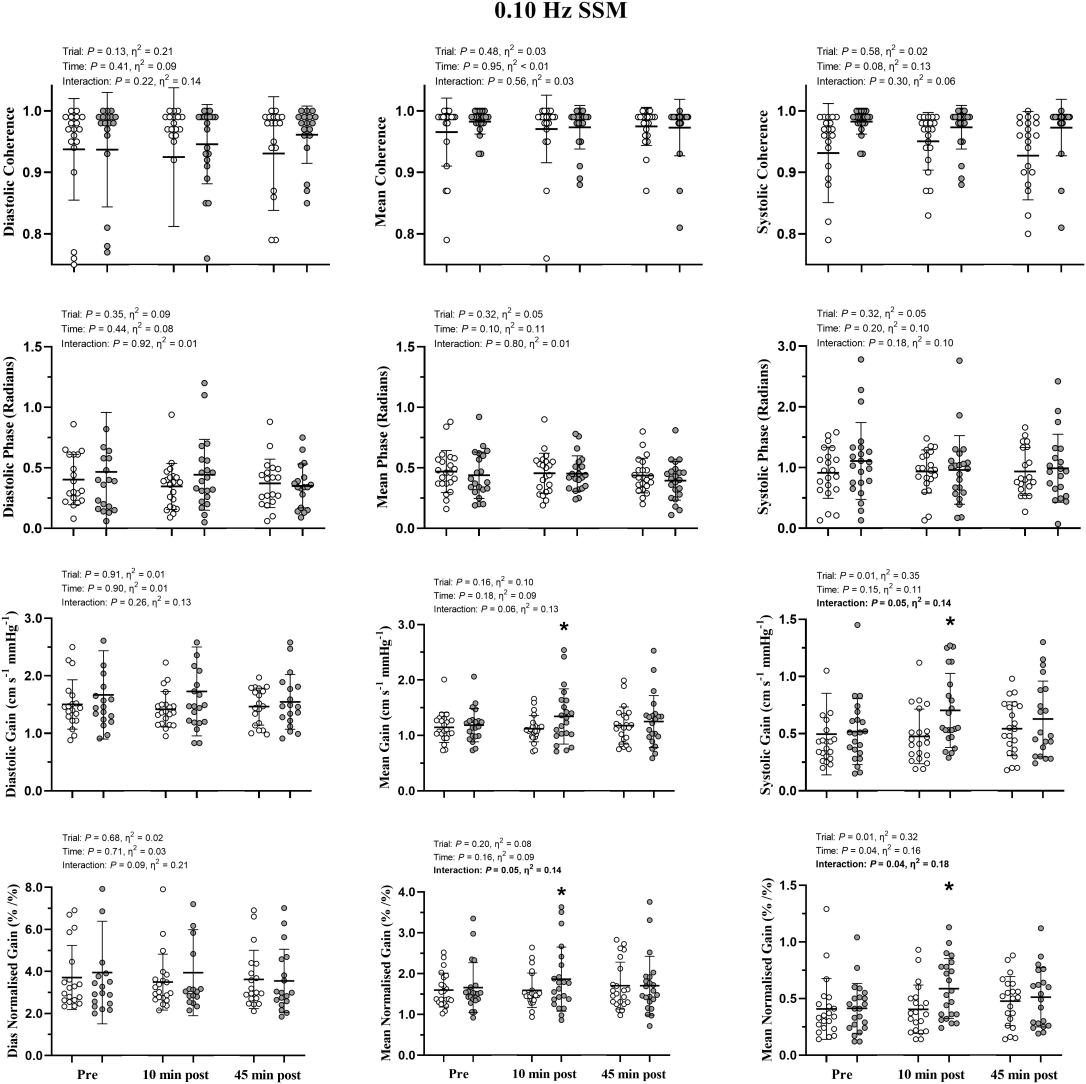
dCA metrics during squat‐stand maneuvers (SSM) at 0.10 Hz preintervention, 10‐ and 45‐minute postintervention. Data are presented as individual values, the mean (horizontal line) and standard deviation (error bars). The control trial (time‐matched seated rest) is presented in white. The gray circles denote the resistance exercise intervention trial (4 sets of 10 repetition back squats at 70% one repetition maximum). Please note the differences in y axes within an outcome, but across the cardiac cycle. Repeated measures ANOVA detected a statistically significant time‐by‐trial interaction for systolic gain, mean normalized gain and systolic normalized gain. * indicates a significant increase compared with pre‐exercise (*p* < 0.02) from ANOVA follow‐up pairwise comparisons.

No significant time‐by‐trial interactions were identified for diastolic phase, gain or normalized gain during 0.05 or 0.10 Hz SSM (*p* > 0.17; η_p_^2^ < 0.28; Figures 3 and 4). Mean and systolic phase, gain and normalized gain also showed no significant time‐by‐trial interactions during 0.05 Hz SSM (*p* > 0.56; η_p_^2^ < 0.07; Figure 3). However, mean gain (*p* = 0.02; *d* = 0.36), systolic gain (*p* = 0.01; *d* = 0.55), mean normalized gain (*p* = 0.02; *d* = 0.28) and systolic normalized gain (*p* = 0.01; *d* = 0.67) were significantly elevated 10‐min postexercise during 0.10 Hz SSM compared with baseline as well as 45‐min postexercise (*p* < 0.05; *d* > 0.18). In contrast, these values were not different to baseline 45‐min postexercise (*p* > 0.16; *d* < 0.15; Figure 4). No significant differences in phase, gain or normalized gain were apparent in the control trial during any phase of the cardiac cycle or at any time point during 0.05 or 0.10 Hz SSM (*p* > 0.10; Figures 3 and 4).

## 4 DISCUSSION

The noteworthy findings of this study are that four sets of 10 repetition back squats at 70% 1RM produced a short‐lived elevation in gain and normalized gain during 0.10 Hz SSM, despite no differences in resting MAP or MCAv after these maneuvers. These changes appear to be driven by a reduction in the ability to buffer BP during systole in young healthy adults.

Our data demonstrate that dCA at 0.10 Hz was altered through an elevation in systolic gain 10‐min postresistance exercise; however, this appears to recover toward baseline within 45 min (Figure 3). There were no significant alterations in P_ET_CO_2_, so it is highly unlikely P_ET_CO_2_ had an influence on the alterations observed in the current findings (Smirl et al., 2015), and the high coherence observed (>0.90) reflects input–output (BP‐MCAv) linearity (Claassen et al., 2016; Zhang et al., 1998). Therefore, our data indicate that there is a brief alteration in the ability to effectively dampen the magnitude of change in MCA_V_ when BP fluctuates during SSM following resistance exercise.

This work extends the previous evidence of an acute impairment in CBF regulation by Koch et al. (2005), which explored dCA only in the seconds immediately following exhaustive resistance exercise (seated leg curling). Additionally, the adoption of two repeated SSM frequencies and assessment of dCA outcomes across the cardiac cycle allows for further inferences to be made in the current study. Specifically, this frequency‐specific (0.10 Hz) change in dCA outcomes indicates altered sympathetic regulation of CBF (Hamner et al., 2010), which is consistent with rodent data demonstrating attenuated cerebrovascular sympathetic innervation following transient hypertension (Phillips et al., 2018). Furthermore, sympathetic mechanisms may be responsible specifically for regulating systolic gain as shown by sympathetic and cholinergic blockade (Hamner et al., 2010; Hamner & Tan, 2014). This is of interest as systolic dCA is typically buffered more effectively than diastolic dCA, potentially as a protective mechanism against cerebral hemorrhage (Smirl et al., 2018). These data are also consistent with the altered systolic dCA metrics observed in young healthy females compared with males during SSM with the addition of ~20% body mass (Newel et al., 2022). Given the widespread advocacy of resistance exercise in public health guidelines, the observed temporary alteration in the ability to buffer against such pressure‐driven alterations in CBF during systole, even in healthy young adults, is worthy of further study.

This acute alteration in dCA is of further interest given that four sets of squats are not necessarily reflective of the totality of a typical strength training session. For example, it is plausible an individual could continue to perform further resistance exercise after these four sets, and thus during a period when dCA is altered. Whether this increases the vulnerability to overperfusion injury or provides an opportunity for positive shear‐stress‐mediated cerebrovascular adaptation (Carr & Ainslie, 2020; Tinken et al., 2010) is unknown and warrants further consideration.

The present data extend our understanding regarding changes in dCA postresistance exercise (Koch et al., 2005; Newel et al., 2022) and highlight a need to further explore the effects of exercise volume, type and intensity. For example, arterial stiffening occurs following high‐intensity, but not moderate‐intensity, resistance exercise (Miyachi, 2013). Our adoption of four sets of 10 repetition squats at 70% 1RM might be considered moderate intensity, although there is little consensus regarding resistance exercise intensity domains. It would be interesting to determine dCA following resistance exercise at higher intensities (80%–100% 1RM), which may better reflect typical training practices in strength athletes (Kraemer & Ratamess, 2004) and may elicit greater BP changes (MacDougall et al., 1985; Newel et al., 2022). For example, our observed alterations in dCA gain might be related to *“weight‐lifters’ blackout”* (Compton et al., 1973), which is thought to reflect a loss in the ability to defend brain blood flow despite alterations in pressure and cardiac output and poses a significant risk to those performing extremely heavy resistance exercise. Our findings also have wider implications for those with pre‐existing risk factors for hemorrhagic stroke (Castro et al., 2018), given that resistance exercise may be advocated for such groups (Pollock et al., 2000), albeit at a lower intensity. We cannot extrapolate our findings beyond young adults, although the influence of age on dCA is currently unclear (Perez‐Denia et al., 2020); however, our data provide a foundation for further exploration into the relationship between acute postexercise alterations in dCA and the longer‐term effects of resistance exercise on CBF regulation. Finally, we note that resistance training is included in Stage 4 of the six‐stage graduated return‐to‐play protocol for athletes recovering from concussion (McCrory et al., 2017). Our observations of a temporary alteration in dCA postresistance exercise in healthy adults suggest that research is needed to inform this recommendation in those with potential cerebral vulnerability.

The fact that neither resting MAP nor MCAv was altered in this study highlights the utility of using complex approaches to understand cerebral pressure‐flow dynamics (Tzeng et al., 2012). However, the novel findings of our paper need to be considered alongside some notable limitations. First, transcranial Doppler ultrasound is unable to directly measure CBF as it determines CBF_V_ (Skow et al., 2022). CBF_V_ provides a robust index of CBF but only if arterial CO_2_ concentrations remain relatively stable to minimize any vasodilatory effects. In line with data presented elsewhere (Smirl et al., 2015; Smirl et al., 2018), the magnitude of changes in P_ET_CO_2_ during the SSM performed pre, 10‐min and 45‐min postexercise in our subsample of 10 participants (due to equipment failure) is unlikely to have elicited vasoactivity. Additionally, our inferences regarding the pressure‐perfusion relationship are driven by blood pressure values recorded at the finger. While this approach is typical of the field (Smirl et al., 2015) and has been utilized to understand any changes in dCA post (Burma, Copeland, Macaulay, Khatra, Wright, & Smirl, 2020b) and during (Newel et al., 2022) exercise, our inferences are vulnerable to any changes in local vascular tone. Second, while our study did not observe any influence of trained status on the dCA response to squats, our study was not specifically powered to determine whether such an interaction effect exists. Whether any chronic adaptation to resistance training (Perry et al., 2019) confers protection from acute alterations in dCA postexercise (Compton et al., 1973) remains an interesting question. So too does a potential interaction effect of sex on any adaptive response (Labrecque et al., 2019), especially given the subtle differences in systolic dCA metrics apparent during light resistance squats recently observed between males and females (Newel et al., 2022). The potential influence of training status and sex remain an interesting area for future study. We also cannot extrapolate our findings beyond young adults, or comment on the responses to heavier loads which are commensurate with strength training. However, our adoption of 10 repetitions is consistent with existing recommendations for health promotion (Fidalgo et al., 2019; Pollock et al., 2000). Finally, only the MCA was insonated in this study. Whether such acute alterations in dCA are observed in the posterior cerebral artery, which supplies ~20% of total CBF (Zarrinkoob et al., 2015), remains to be assessed.

## 5 CONCLUSION

We observed that gain and normalized gain at 0.10 Hz were significantly elevated 10‐min postresistance exercise, and this appears to be driven by alterations in pressure‐flow dynamics during systole. Such changes appear to be short‐lived, with all dCA metrics returning to baseline within 45 min. These findings suggest that sympathetic regulation of dCA can be transiently reduced postresistance exercise. Further research exploring the effects of exercise volume, type and intensity is warranted to elucidate the relationship between resistance exercise and dCA, as well as a deeper exploration of continued resistance exercise during this 10‐minute window of potential vulnerability.

## AUTHOR CONTRIBUTIONS

OS and BB conceived the study, which was then designed by OS, BB, JS, and AB. OS, QAA, DC, and WW completed data collection. OS, QAA, DC, WW, and BB performed data analysis. All authors contributed to data interpretation. OS and BB drafted the initial manuscript, which was then edited by all and subsequently revised. All authors approved the final manuscript.

## FUNDING INFORMATION

This project was not externally funded.

## CONFLICT OF INTEREST STATEMENT

The authors have no competing interests to declare that are relevant to the content of this article.

## ETHICS STATEMENT

Ethics approval was granted by the institutional ethics committee prior to the commencement of this work (reference number: 2019/M/10). All participants provided written, informed consent before taking part. The datasets analyzed during the current study are available from the corresponding author on reasonable request. The authors have no conflicts of interest to disclose. This work was not supported by external funding.
